# The genetic validation of heterogeneity in schizophrenia

**DOI:** 10.1186/1744-9081-7-43

**Published:** 2011-10-07

**Authors:** Atsushi Tsutsumi, Stephen J Glatt, Tetsufumi Kanazawa, Seiya Kawashige, Hiroyuki Uenishi, Akira Hokyo, Takao Kaneko, Makiko Moritani, Hiroki Kikuyama, Jun Koh, Hitoshi Matsumura, Hiroshi Yoneda

**Affiliations:** 1 Department of Neuropsychiatry; Osaka Medical College; Osaka, Japan; 2 Department of Psychiatry and Behavioral Sciences, and Medical Genetics Research Center; SUNY Upstate Medical University; 750 East Adams Street; Syracuse, NY, 13210, USA; 3 Graduate School of Letters, Kansai University; Osaka, Japan; 4 Laboratory of Pharmacotherapy; Osaka University of Pharmaceutical Sciences; Osaka, Japan

**Keywords:** schizophrenia, gene, Schizophrenia Gene Database (SZGene), heterogeneity, Japanese, DRD2, DRD4, GRIN2B, TPH1, MTHFR, DTNBP1, and Risk-Index

## Abstract

**Introduction:**

Schizophrenia is a heritable disorder, however clear genetic architecture has not been detected. To overcome this state of uncertainty, the SZGene database has been established by including all published case-control genetic association studies appearing in peer-reviewed journals. In the current study, we aimed to determine if genetic variants strongly suggested by SZGene are associated with risk of schizophrenia in our case-control samples of Japanese ancestry. In addition, by employing the additive model for aggregating the effect of seven variants, we aimed to verify the genetic heterogeneity of schizophrenia diagnosed by an operative diagnostic manual, the DSM-IV.

**Methods:**

Each positively suggested genetic polymorphism was ranked according to its p-value, then the seven top-ranked variants (p < 0.0005) were selected from DRD2, DRD4, GRIN2B, TPH1, MTHFR, and DTNBP1 (February, 2007). 407 Schizophrenia cases and 384 controls participated in this study. To aggregate the vulnerability of the disorder based on the participants' genetic information, we calculated the "risk-index" by adding the number of genetic risk factors.

**Results:**

No statistically significant deviation between cases and controls was observed in the genetic risk-index derived from all seven variants on the top-ranked polymorphisms. In fact, the average risk-index score in the schizophrenia group (6.5+/-1.57) was slightly lower than among controls (6.6+/-1.39).

**Conclusion:**

The current work illustrates the difficulty in identifying universal and definitive risk-conferring polymorphisms for schizophrenia. Our employed number of samples was small, so we can not preclude the possibility that some or all of these variants are minor risk factors for schizophrenia in the Japanese population. It is also important to aggregate the updated positive variants in the SZGene database when the replication work is conducted.

## Introduction

Schizophrenia is a highly heritable disorder, but even its genetic architecture still remains unclear [1]. In order to reveal the genetic involvement in the etiology, several research designs have been adopted. Linkage studies have not, however, found strongly linked loci [2]. Alternatively, many association studies of schizophrenia have been performed because genetic association methods are a more powerful means for finding genes that are expected to have a small effect on risk for the disorder. Although many positive results were reported, most are never replicated extensively. To overcome this state of uncertainty, Bertram et al. assembled the SZGene database to aggregate the evidence for those genetic polymorphisms with the best, most reliable evidence for association with schizophrenia [3,4]. SZGene is characterized by the inclusion of all published case-control genetic association studies appearing in peer-reviewed journals, and each association is evaluated using a uniform method of meta-analysis. This inclusive and consistent database renders sometimes ambiguous or amorphous bodies of evidence concrete, and highlights some of the most promising avenues for continued research by schizophrenia geneticists. From this database, we picked the most reliably associated variants, and sought to determine if these variants would be associated with risk for schizophrenia in our own case-control sample of Japanese ancestry. Consistent with a common-disease/common-variant hypothesis of the disorder, we would not hypothesize that each variant identified in SZGene would be a necessary component of the risk profile for schizophrenia in our sample, so we evaluated the independent and joint effects of the top seven variants. The objectives of this study were: 1) to determine if the top candidate genes for schizophrenia would show replicated evidence for a contribution to risk for the disorder; and 2) to see if the aggregate effect of these seven top variants was stronger than the individual effects of each variant alone.

### Methods

Based on the information in SZGene as of February 2007, each positively associated genetic polymorphism in the database was ranked according to its p-value from a chi-square test of association with the disorder. The p-values of the seven top-ranked polymorphisms were all less than 0.005 (Table 1). We chose these seven variants for the pragmatic reason of cost and effort. To evaluate these selected polymorphisms, we recruited 407 patients diagnosed with schizophrenia by multiple psychiatric doctors according to DSM-IV TR criteria and 384 normal controls without any medical record in psychiatry. Every participant gave written and oral informed consent, and this study was approved by the ethical committee at Osaka Medical College. The cases were recruited from inpatients in two mental hospitals and one university hospital located in the western area of Japan, and the controls were recruited among hospital staff. The details of the samples are described in the Table 2. No statistical difference was observed in the male/female ratio of the participants, while the patients were significantly younger than the controls. All of the participants lived in the western area of Japan, and were self-reported ethnically Japanese.

**Table 1:** Derived data from SZGene for investigated genes and polymorphisms

	Gene	Polymorphism	Denomination	p-value	OR	95%CI	Cases				Control			
							
							m/m	m/M	M/M	Sum	m/m	m/M	M/M	Sum
1	DRD2	rs6277(C/T)		5.71.E-06	1.4	(1.2-1.63)	178	266	130	574	225	484	332	1041

2	DRD2	rs1801028(G/C)		8.24.E-06	1.34	(1.11-1.61)	9	270	3770	4049	8	254	5420	5682

3	GRIN2B	rs1019385(T/G)	(200T/G)	4.70.E-05	0.69	(0.54-0.88)	85	256	161	502	114	257	95	466

4	TPH1	rs1800532(A/C)		7.99.E-05	1.25	(1.08-1.44)	324	609	306	1239	344	851	513	1708

5	MTHFR	rs1801133(T/C)	(C677T)	2.15.E-04	1.14	(1.03-1.25)	490	1672	1708	3870	550	2265	2536	5351

6	DTNBP1	rs2619528(A/G)	(P1765)	4.08.E-04	1.25	(1.01-1.56)	90	558	1049	1697	79	641	1485	2205

7	DRD4	120-bpTR(S/L)		3.80.E-03	0.81	(0.7-0.94)	55	374	807	1236	77	412	710	1199

8	PLXNA2	rs1327175(G/C)	0.043	0.76	(0.58-0.99)	12	221	1478	1711	15	280	1475	1770	

9	DAOA	rs3916971(T/C)	0.045	0.84	(0.73-0.96)	169	401	274	844	213	457	252	922	

10	HP	HP1/2		0.044	0.88	(0.8-0.98)	184	647	515	1346	324	995	699	2018

Abbreviation:

DRD2 = dopamine receptor D2, GRIN2B = glutamate receptor, ionotropic, N-methyl D-aspartate 2B, TPH1 = tryptophan hydroxylase 1, MTHFR = methylenetetrahydrofolate reductase, DTNBP1 = dystrobrevin binding protein 1, DRD4 = dopamine receptor D4, PLXNA2 = plexin A2, DAOA = D-amino acid oxidase activator, HP = haptoglobin

SCZ = Schizophrenia, NCs = Normal Controls, rs = Reference Single Nucleotide Polymorphism, bpTR = base pair Tandem Repeat, m = wild allele, M = mutant allele, S = Short allele, L = Long allele, SZGene = Schizophrenia Gene Database (http://www.szgene.org/), OR = Odds Ratio, CI = Confidential Interval

**Table 2:** Demographic Samples

	Number	Male	Mean Age	PANSS Average	Paranoid form	Hebephrenic form	Catatonic form
SCZ	407	221(54.3%)	47.2	80.2	76.4%	13.5%	3.1%

NCs	384	194(50.5%)	42.1	-	-	-	-

Abbreviation:

SCZ = Schizophrenia, NCs = Normal Controls, PANSS = Positive and Negative Syndrome Scale

With regard to the genotyping procedure, Single nucleotide polymorphisms (SNPs) on the genomic DNA extracted from venous blood were identified by FRET method (Roche Diagnostics, Japan) according to the manufacture's protocol. The 120 bp Tandem Repeat polymorphism in DRD4 was detected by restriction fragment length polymorphism. Each primer and probe was provided on demand.

In addition to the analysis of each single polymorphism, we sought to aggregate the vulnerability of the disorder based on the participants' genetic information. The risk alleles of each of the seven top polymorphisms shown in SZGene were summated, such that an individual homozygous for a polymorphism's risk alleles was quantified as having a value of "2". The heterozygous genotype was given the value of "1", and the homozygote of non-risk alleles was coded "0". This scoring imparted an additive effect onto each variant, which was supported by the meta-analytic results in SZGene (although this may not be the true mode of inheritance of each gene's effect on risk, misspecification by an additive model is less costly than misspecification by other models). Each of the participants was additively scored from 0 to 14 according to their genetic vulnerability information from across all seven variants. This score was then entered as a predictor of case status in a logistic regression model, with the type-I-error rate fixed at 0.05.

## Results

**Figure 1:**
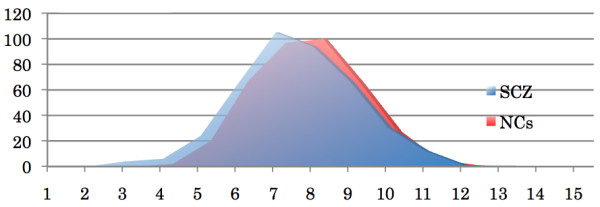
**Numbers of the samples with each Risk-Index**. X-axis is "Risk Index", and Y-axis is the Numbers of the samples. The average and standard deviation of "Risk Index" for schizophrenia was 6.5 ± 1.57, while 6.6 ± 1.39 for normal controls. Abbreviation: SCZ = Schizophrenia, NCs = Normal Controls

The distribution of no single polymorphism was significantly different between schizophrenia and control groups at a p-value less than 0.05 (Fisher's exact test for each variant and t-test for aggregated seven variants, Table 3). Moreover, no statistically significant deviation in the genetic vulnerability index derived from all seven variants was found within the seven top-ranked polymorphisms. In fact, the average risk-index score in the schizophrenia group (6.5 ± 1.57) was slightly lower than among controls (6.6 ± 1.39) (Figure 1). This is consistent with the negative results from the logistic regression analysis of the risk-index score, which found a non-significant (p = 0.783) and slightly negative (β = -0.02) effect of increments in the risk-index on likelihood of being in the schizophrenia group. Our failure to find a higher-order genetic effect of these seven variants on vulnerability to schizophrenia persisted when we reanalyzed the data after assigning a weight to each risk allele proportional to its odds ratio as indicated in SZGene. Again, a negative result was obtained when risk-allele carriers (risk-allele homozygotes and risk-allele heterozygotes) were pooled, suggesting that neither an additive nor a dominant model of the joint effects of these seven variants could be supported.

**Table 3:** P-value for the Individual SNPs and the Aggregation of Seven Variants

Gene	SNP	SCZ			NCs			Individual SNPs(Fisher's exact test)	Aggregation of Seven SNPs(t-test)
								**p-value for Additive effect**	**p-value for Additive effect**

DRD2	rs1801028	**C/C**	**C/G**	**G/G**	**C/C**	**C/G**	**G/G**	0.275	
	
		390	14	8	354	23	7		
	
DRD2	rs6277	**C/C**	**C/T**	**T/T**	**C/C**	**C/T**	**T/T**	0.362	
	
		367	38	1	341	43	2		
	
GRIN2B	rs101938	**T/T**	**T/G**	**G/G**	**T/T**	**T/G**	**G/G**	0.67	
	
		91	206	110	81	204	100		
	
TPH1	rs1800532	**A/A**	**A/C**	**C/C**	**A/A**	**A/C**	**C/C**	0.635	0.189
	
		105	219	90	108	200	78		
	
MTHFR	rs1801133	**T/T**	**T/C**	**C/C**	**T/T**	**T/C**	**C/C**	0.519	
		69	184	160	64	183	138		
	
DTNBP1	rs2619528	**G/G**	**G/A**	**A/A**	**G/G**	**G/A**	**A/A**	0.193	
	
		326	77	4	323	60	1		
	
DRD4	120bpTR	**S/S**	**S/L**	**S/L**	**S/S**	**S/L**	**L/L**	0.095	
	
		24	138	248	16	158	211		

## Discussion and Conclusion

In this study, we utilized the evidence from the SZGene database to build very strong hypotheses of independent and joint effects of the top seven most strongly supported risk genes for schizophrenia. These hypotheses were roundly unsupported. While our results alone are not enough to invalidate the conclusions gleaned from the large body of evidence collated in SZGene, they do cast doubt about the strength of the documented associations. Several potential confounders may be able to explain our results.

Chiefly, our failure to find significant associations could be a result of insufficient sample size and less-than-optimal statistical power. Our average power was 6.72% on the assessment of Genetic Power Calculator (type-I error rate at 0.05, and the prevalence of the disorder at 0.008, http://pngu.mgh.harvard.edu/~purcell/gpc/, Table 4). This level of power is not enough to confidently refute the claims of significant association for these seven variants. The minor allele frequencies of some of these seven variants (in particular, rs1801028 on *DRD2*) are quite low (e.g., f = 0.05), which has adverse effects on the inferential power to detect the effects of those variants. Furthermore, this negative impact on power is likely to be compounded when investigating the joint effects of the seven SNPs. However, the direction of the effects identified in our sample was, for five of the variants (rs1801028 on *DRD2*, rs1019385 on *GRIN2B*, rs1800532 on *TPH1*, rs2619528 on *DTNBP1*, and rs1801133 on *MTHFR*), in the opposite direction of that documented in SZGene. Thus, even with the addition of more subjects, our sample likely would not yield any semblance of significant association for these five variants. For the remaining two variants (120bpTR on *DRD4 *and rs6277 on *DRD2*) our detected effects were in the same direction as those reported in SZGene, suggesting that our data may in fact be consistent with the hypothesis of some effect of these variants on risk for the disorder in a global sense (though not necessarily a significant effect in our sample).

**Table 4:** The deviation from Hardy-Weinberg Equilibrium (*X*^2^, more than 3.84 represents the significant deviation from HWE at 0.05 p-value) and genetic power for investigated samples (prevalence = 0.008, type II error = 0.80, general 2df, alpha = 0.05)

		SCZ	NCs	Power
DRD2	rs1801028	0.67	4.16	0.0504

DRD2	rs6277	109.57	46.21	0.0919

GRIN2B	rs101938	0	0.26	0.0525

TPH1	rs1800532	0.09	1.5	0.0806

MTHFR	rs1801133	1.46	0.7	0.0563

DTNBP1	rs2619528	1.66	0.06	0.069

DRD4	120bpTR	0.05	1.07	0.0698

Another caveat in interpreting our results regards the analytic models we employed. In the present study, in order to reduce the complexity, it was simply hypothesized that the attributable odds ratio for each polymorphism was 2, and we hypothesized that vulnerability could be calculated additively (however, we also evaluated a dominant model with similar negative results). It could be suspected that the mode of inheritance of schizophrenia is not simply additive, however no definitive model is identified so far [1,5,6]. Re-analyses of these data with other modes of inheritance specified (e.g., recessive, multiplicative) may have yielded different results more consistent with expectations based on SZGene.

A third caveat regarding interpretation of our study is the ancestry of our sample. All of our subjects were derived from a sample of Asian ancestry, Japanese, although the top seven variants were selected based on their collective evidence from all ancestral groups (though SZGene represents more Caucasian samples than Asian ones). Given the possibility that the current lack of detection derived from this discrepancy, we recalculated the top variants in SZGene from samples of only Asian ancestry. Within the seven variants, the majority of variants (four out of seven) were still significant in the Asian-only subsamples, and the other three variants had only been examined in Asian-ancestry samples once or twice previously, precluding definitive answers. These data imply that the investigated variants were deserving of further research both in general and in Asian samples specifically. Nevertheless our currently employed samples will not generalize to all samples evaluated by the schizophrenia geneticists so far.

With regard to the deviation from Hardy-Weinberg Equilibrium (HWE), the current result of genotyping in cases for 120bp Tandem Repeat in DRD4, and both in cases and controls for rs1801028 in DRD2 has shown the deviation from HWE (Table 4). Our finding on this article is not the simple positive association, therefore these deviations will not have a major influence on our overall conclusion. Although it is possible to expect that the deviated three group have false-negative association, we still conclude that our main finding will not be forced to change the fundamental direction.

Given these potential confounders it is keenly necessary to repeat this investigation in a larger, ethnically diverse population in order to validate (or invalidate) our model further. It is also important to aggregate the updated positive variants in the SZGene database when the replication work is conducted. Much work is needed in a collaborative effort in order to generate power to identify potential genetic markers for schizophrenia that transcend ethnicities and populations worldwide, if such exist. Studies of schizophrenia inevitably inherit the weaknesses of the current psychiatric nosology, which clinical observation (symptomatology) demands when we aimed to select the sample due to the lack of biological markers. Our current findings that there is no statistically significant deviation in the possession of "risk-conferring" alleles between schizophrenia and controls may have a chance to highlight the profound heterogeneity of the disorder of interest.

## Competing interests

The authors declare that they have no competing interests.

## Authors' contributions

TK and SJG designed the study and collaborated in the writing of the final version of the manuscript. SK, AT, and HK wrote the protocol, and managed the genotyping. SK wrote the first draft. HU and SJG undertook the statistical analysis. AH, TK, MM, and JK worked for the sample recruitment. HM, and HY assisted in conceptualizing the study, provided laboratory space and resources for data collection, and collaborated in the writing of the final version of the manuscript. All authors contributed to and have approved the final manuscript.
